# Examining the Constructs of Burnout, Compassion Fatigue, Secondary Traumatic Stress in Physicians Using Factor Analyses

**DOI:** 10.3389/fpubh.2022.893165

**Published:** 2022-05-06

**Authors:** Fadwa Ahmed, Jay Baruch, Paul Armstrong

**Affiliations:** ^1^Warren Alpert Medical School of Brown University, Providence, RI, United States; ^2^Department of Emergency Medicine, Warren Alpert Medical School of Brown University, Providence, RI, United States; ^3^Department of English, Brown University, Providence, RI, United States

**Keywords:** burnout, compassion fatigue, secondary traumatic stress, physicians, factor analysis

## Abstract

**Background:**

Adverse affective experiences have been well-documented in healthcare providers. Research describes them under a variety of terms, including burnout, secondary traumatic stress (STS), and compassion fatigue (CF). The present study evaluates conflicting models of STS, CF, and burnout constructs in physicians.

**Methods:**

Surveys were mailed to all allopathic physicians with active Rhode Island medical licenses. Three hundred and seventy-five complete responses were received. The survey included common measures of STS, CF, and burnout. Confirmatory Factor Analysis (CFA) was used to evaluate discriminant validity of the three constructs and test 5 *a priori* (1-, 2-, and 3-factor) theoretical models, and Exploratory Factor Analysis (EFA) was planned assess underlying factor structure in the case that CFA did not provide evidence supporting any existing model.

**Results:**

By CFA, all five *a priori* models of burnout, CF, and STS fail to demonstrate adequate model fit (Standardized Root Mean Square Residual >0.10, Tucker-Lewis Index <0.90). EFA with parallel analysis extracts four factors underlying the three burnout, STS, and CF measures. The four factors describe 54.3% of variance and can be described as (1) depressive mood; (2) primary traumatic stress-like symptoms; (3) responses to patients' trauma; and (4) sleep disturbances.

**Conclusion:**

In spite of abundant discussion surrounding burnout, CF, and STS in physicians, measures of these constructs did not uphold their theoretical factor structures in the present study. Future research might explore other constructs and measures that may describe adverse affective physician experiences.

## Introduction

Work-related adverse experiences have been well-documented in populations of healthcare providers. Research of these adverse effects describes them under a variety of terms, the most prominent of which include secondary traumatic stress (STS), compassion fatigue (CF), and burnout. Although they are frequently discussed together in relation to physicians, there has been little agreement about the differences and relationships between STS, CF, and burnout in this population.

*Burnout* is generally used to describe work-induced exhaustion and demotivation that can affect one's ability and willingness to do their work (1–3). Often, it is regarded as a multidimensional construct, containing elements of physical exhaustion, emotional and spiritual disturbance, depersonalization, and a reduced sense of personal fulfillment (4–7). Though burnout is generally defined as a function of work-related and professional factors, the distinction between burnout and non-work-related syndromes such as depression remains a subject of contention (8).

Charles Figley's early descriptions of *secondary traumatic stress* (STS) define it as the “stress from helping or wanting to help a traumatized person” (9). More recent literature has defined it as a state similar to Post-Traumatic Stress (PTS) that is induced by secondary, rather than primary, exposure to trauma (10). Analogous to burnout and depression, PTS and STS have been regarded as similar but distinct phenomena, their primary difference pertaining to their etiological factors (11).

*Compassion fatigue* (CF) describes some broader range of negative affective experiences that results from attempts to assist others undergoing difficulty (12, 13). Most definitions of CF in physicians refer to a healthcare provider's loss of capacity to engage compassionately with patients (14).

Research has shown that CF, STS, and burnout correlate with each other, and whether or not they constitute fundamentally distinct phenomena remains unclear (15–17). In Figley's initial model, he regards CF and STS as interchangeable terms, defining burnout as a distinct but related phenomenon (9). Other literature has instead proposed that CF represents a syndrome that arises from the co-occurrence of burnout and STS and contains symptoms of both (18, 19). The Professional Quality of Life Scale, perhaps the most widely used of any CF measure, models CF as a composite of burnout and STS symptoms (12).

Several other authors, however, propose that CF, STS, and burnout represent distinct phenomena (14, 20). A number of theories point to differences in etiology, time course, influencing factors, and qualitative attributes as distinguishing features. For instance, CF and STS have been distinguished based on the differential importance of risk factors such as provider empathy and exposure to patients' trauma (20, 21). Similarly, burnout and compassion fatigue, while sometimes regarded as synonyms (22, 23), have been differentiated in terms of onset and time course (24–26) as well as the circumstances that lead to them (14, 27–29). Some have regarded burnout as a precursor to compassion fatigue, and others vice versa (14, 24, 30–35). Others still suggest the absence of a strong correlation between the two (21, 36).

**Figure 1:**
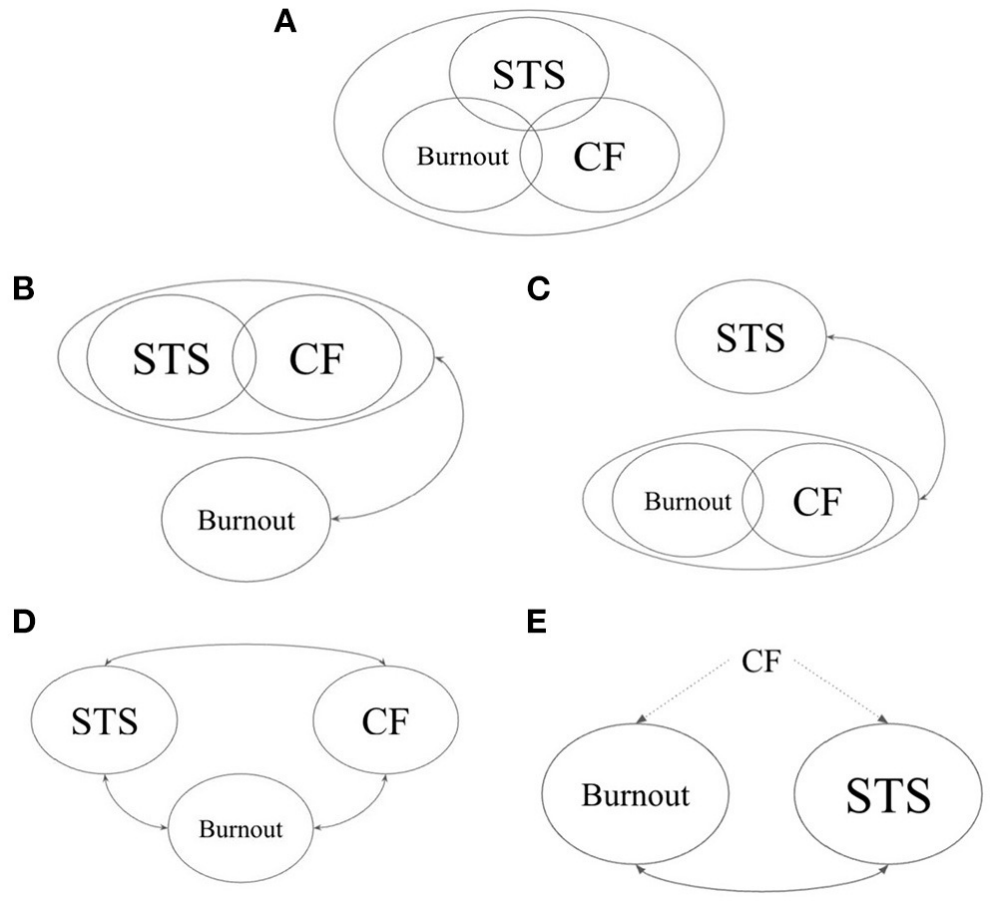
*A-priori* models tested in confirmatory factor analyses. **(A)** Model 1: STS, CF, and burnout represent a single underlying construct (37). **(B)** Model 2: STS and CF represent a single underlying construct, while burnout represents a separate and correlated construct (9). **(C)** Model 3: Burnout and CF represent a single underlying construct, while STS represents a separate and correlated construct (22, 23). **(D)** Model 4: STS, CF, and burnout each represent a separate construct (38). **(E)** Model 5: CF describes a bifactorial construct in which burnout and STS are distinct but correlated factors (12). STS, secondary traumatic stress; CF, compassion fatigue.

Considering the variety of terms and models used to describe negative affective experiences among physicians, the extent to which each represents its own distinct construct remains unclear. The present study seeks to clarify the discriminant validity and factor structure of burnout, CF, and STS constructs. Using cross-sectional survey data from a sample of Rhode Island physicians, the present study seeks to evaluated whether confirmatory factor analysis (CFA) provides supporting evidence to any of the multiple conflicting models of burnout, CF, and STS (9, 12, 22, 23, 37, 38) (Figure 1).

In the case that CFA did not support any existing models, we planned to conduct an exploratory factor analysis (EFA) to gain insight into the factor structure underlying current measures of burnout, CF, and STS.

## Methods

### Study Sample and Recruitment Procedure

For this study, surveys were distributed to Rhode Island physicians both by paper mail and electronically between July and August 2019. A sample size of at least 300 participants was sought to achieve adequate power for factor analyses (39). A list of all allopathic physicians with full, active Rhode Island licenses was obtained through the Rhode Island Department of Health (RIDOH) online database. Surveys were sent *via* postal mail to all physicians with mailing addresses listed in the database (*N* = 3,598). An online version of the survey hosted on Qualtrics survey software was also sent to all physicians with e-mail addresses listed in the RIDOH database (*N* = 1,877). Confidentiality was maintained and participation was voluntary. The survey was sent to a total of 3,598 physicians, 387 of whom were ineligible because they were not at the listed address, deceased, or no longer active in patient care, yielding an eligible sample of 3,211. Responses were collected through October 2019. The institutional review board of Brown University approved this study and waived the requirement for written informed consent, as participation was voluntary and completion of the survey implied consent. This study followed Strengthening the Reporting of Observational Studies in Epidemiology (STROBE) guidelines.

### Measures

In addition to questions pertaining to demographic and medical practice information, the survey included self-report measures for burnout, STS, and CF, selected based on their widespread use and strong prior demonstrations of unidimensional construct validity.

#### Secondary Traumatic Stress Scale

The measure consists of 17 items, and prompts responders to answer based on how frequently each item statement was true for them in the prior 7 days (40). Answers range from “Never” to “Very Often” on a five-point Likert scale. Several items refer directly to work-related experiences with “client(s)” in whom responders are in “helping relationships with.” In this study, the word “clients” was replaced with “patients.” The measure has high internal consistency (α = 0.89) and consists of three subscales: Intrusion (α = 0.68), Avoidance (α = 0.78), and Arousal (α = 0.76). Subscales were not analyzed separately in the present study, since the unified construct of STS was the variable of interest.

#### Burnout Measure, Short Version

The scale is a 10-item, short version of the Burnout Measure (4) with high internal consistency (α = 0.90) (2). Each item is a continuation of the statement “When you think about your work overall, how often in the last month have you felt…”, with answers on a seven-point Likert scale ranging from “Never” to “Always.” It assesses physical, mental, and emotional exhaustion to evaluate burnout and is a satisfactory measurement of burnout as a unidimensional construct (2, 13).

#### Professional Quality of Life, Revised (ProQOL-21)

This is a revised version of the ProQOL (12) with 21-items scored on three-to-five point Likert scales, depending on the item (13). It is composed of two subscales that offer a more robust measurement of Compassion Satisfaction (CS; 10 items; α = 0.88) and CF (11 items; α = 0.80). Heritage, Rees, and Hegney (13) modified the original ProQOL such that the CF subscale more precisely targets a unidimensional construct. Though the CF and CS items are included together on the ProQOL-21, the two subscales target separate constructs, and scores for each subscale are to be interpreted separately rather than added to produce a total score. Only the CF subscale of the ProQOL-21 was of interest in the present study. To facilitate interpretation, the items of the ProQOL-21 are labeled with their original ProQOL item numbers throughout this article. The ProQOL-21 was selected over the original due to its robust demonstration of a unidimensional CF construct, which was the variable of interest in the present analysis.

### Statistical Analyses

Since several theories exist regarding the factor structure and measurement properties of burnout, CF, and STS, CFA was deemed to be the most appropriate initial approach to evaluate these theories (41–43). CFA evaluates whether observed variables (data obtained through measurement scales) are linked to higher-order latent factors as postulated in theoretical models, and is a more appropriate initial approach than EFA in cases where such *a priori* models exist (42). Five *a-priori* models, based on the several proposed theories pertaining to burnout, CF, and STS, are tested using CFA in the present study (See Figure 1 for graphical depictions of each model).

In Models 1–4, CF, STS, and burnout each represented a latent variable onto which all items from their associated scales (ProQOL-21 CF subscale, STSS, and BM-SV, respectively) were permitted to load onto. In all models, latent variable means and variances were standardized to zero and one, respectively. In Model 1, covariances between latent Burnout, CF, and STS factors were all constrained to one and thus represented a single underlying latent factor (37). In Model 2, only the covariance between CF and STS was constrained to one (9), and in Model 3, only the covariance between latent CF and burnout factors was constrained to one (22, 23). Model 4 tests burnout, CF, and STS as three distinct latent factors permitted to covary freely with one another (38).

Given that the original ProQOL was designed based on the premise that CF represents a bifactorial construct consisting of burnout and STS (12), a fifth model (Model 5) was tested in which STS and burnout each represent a latent variable. STSS items and STS-specific ProQOL-21 items permitted to load onto the former, and BM-SV items and burnout-specific ProQOL-21 items permitted to load onto the latter.

Because the data was ordinal and observations demonstrated non-normal distributions, a diagonally weighted least square (DWLS) approach was used to estimate model parameters, and the full weight matrix used to compute robust standard errors and a mean- and variance-adjusted test statistic (44). Assessment of model fit was based on interpretation of multiple fit indices, bearing in mind the following conventional criteria of good fit: χ^2^/d.f. ≤ 3, Tucker-Lewis Index (TLI) ≥ 0.95, Root Mean Square Error of Approximation (RMSEA) ≤ 0.08, and Standardized Root Mean Square Residual (SRMR) <0.10. Because Models 1 through 4 were nested, these models were compared to one another using the χ^2^ difference test (45). In the absence of a well-fitting CFA, we planned to perform an EFA using principal axis factoring (PAF).

Because all variables had <2% missing data, and because fewer than 5% of participants had missing data, these missing data were deemed negligible and only complete cases were included in final analyses. Sensitivity analyses were performed including incomplete cases, with missing data imputed using multiple hot deck imputation across five datasets.

Hot deck imputation and CFA was conducted in R version 4.1.1 using the hot.deck package version 1.2 (46) and lavaan latent variable analysis package version 0.6.8 (47), respectively. All other statistics were performed using SPSS 28.0. SPSS syntax provided by O'Connor (48) were used to run Velicar's minimum average partial test and parallel analyses to determine the number of factors to retain for EFA.

## Results

### Sample Characteristics

A total of 270 paper and 135 electronic responses were recorded, 16 of which did not contain answers to any items on relevant burnout, CF, and STS scales and were thus excluded as non-responses. Of a remaining total of 389 responses, 13 were missing between 1 and 10 responses for individual items on relevant scales (2.6–24.6%) and one was missing 36 items (94.7%). These 14 partial responses were excluded, leaving a total of 375 complete responses included in the final analysis, 265 (70.7%) postal mail and 110 (29.3%) electronic responses. The calculated response rate (American Association for Public Opinion Research equation RR3) was 20%. Demographic and professional characteristics of participants are listed in Table 1.

**Table 1:** Demographic and professional characteristics of physician survey responders included in final analysis (*N* = 375).

**Characteristic**	**No. (%)**
Age, mean (SD), y	52.43 (11.70)
Female	159 (42.4)
**Race/ethnicity**	
White	298 (79.5)
Black	6 (1.6)
Hispanic/Latino	17 (4.5)
Asian	37 (9.9)
Other/blank	17 (4.5)
Years in practice, mean (SD)	24.1 (12.2)
**Practice setting** ^**a**^	
Hospital-based	162 (42.6)
Large group practice (6+ physicians)	96 (25.3)
Small group practice (<6 physicians)	64 (16.8)
Individual/solo practice	39 (10.3)
Other^b^/blank	30 (7.9)
**Role** ^**a**^	
Attending physician/hospitalist	257 (66.1)
Head of department	31 (9.0)
Medical director	71 (18.3)
Fellow	9 (2.3)
Unspecified	26 (6.7)
**Specialty, no. (%)**	
Anesthesiology	9 (4.8)
Dermatology	5 (1.3)
Emergency medicine	12 (3.2)
Family/general practice	26 (6.9)
Internal medicine	91 (24.3)
Neurology	9 (2.4)
Obstetrics/gynecology	19 (5.1)
Ophthalmology	11 (2.9)
Otolaryngology	5 (1.3)
Pathology	7 (1.9)
Pediatrics	59 (15.7)
Psychiatry	49 (13.1)
Radiology	5 (1.3)
Surgery	28 (7.5)
Surgical subspecialty^c^	10 (2.7)
Other^d^/missing	30 (8.0)

a *Numbers add up to >100% because some reported more than one setting and/or role*.

b *Includes community health centers, federally-qualified health centers, and urgent care*.

c *Neurological, plastic, and orthopedic surgery*.

d *Includes clinical genetics, urology, physical medicine and rehabilitation, and occupational medicine*.

*No., number; y, years; SD, standard deviation*.

### Confirmatory Factor Analyses

**Figure 2:**
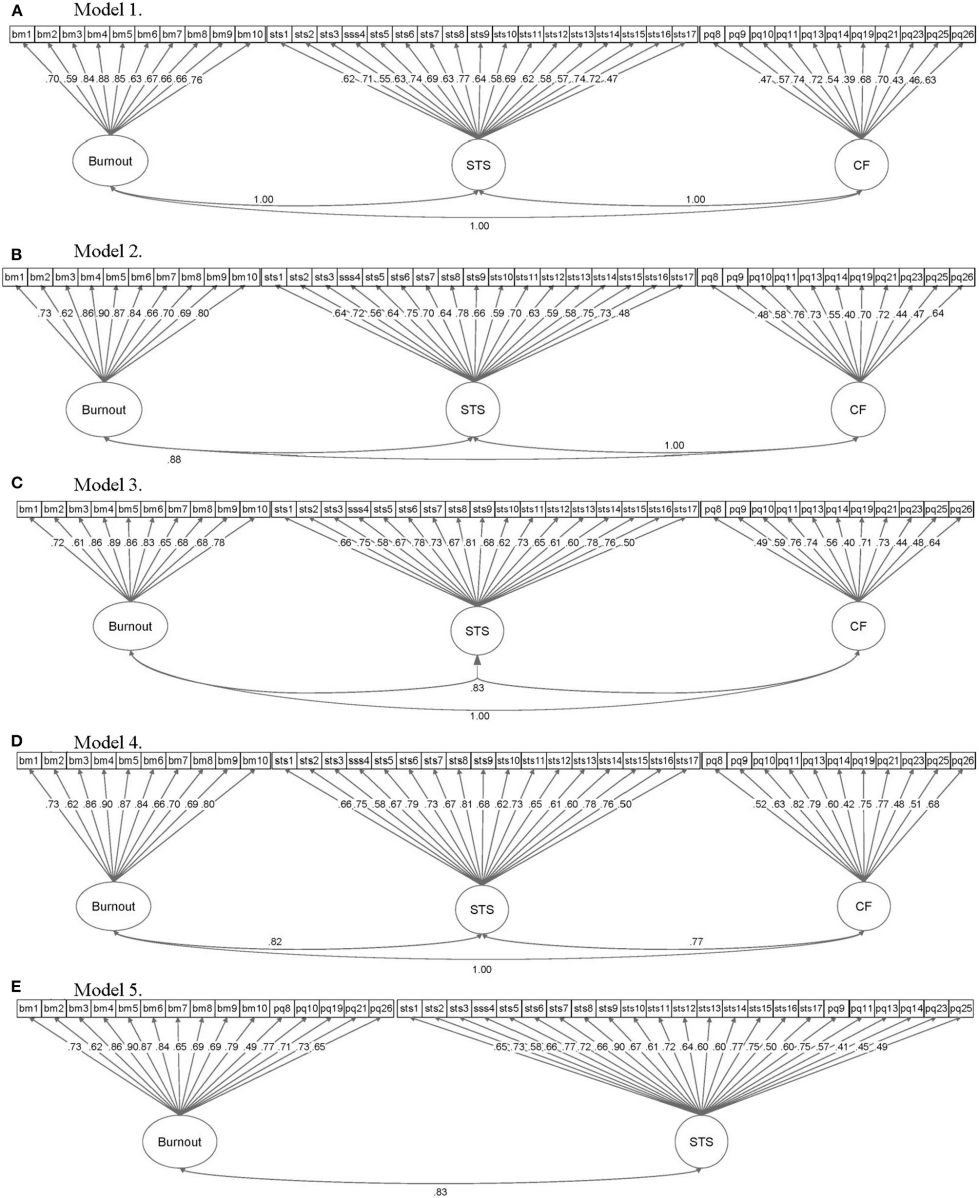
Graphical summary of confirmatory factor analyses results for Models 1–5 of burnout, compassion fatigue, and secondary traumatic stress. Elliptical objects represent latent variables, while rectangular objects represent observed variables (i.e., survey scale items). CF, compassion fatigue; STS, secondary traumatic stress; bm, Burnout Measure-Short Version; pq, ProQOL (although the ProQOL-21 was used, item numbers correspond to original ProQOL item numbers); stss, Secondary Traumatic Stress Scale.

All *a-priori* models of burnout, compassion fatigue, and secondary traumatic stress failed to demonstrate adequate model fit in CFA. Model fit indices are summarized in Table 2 and Figure 2 contains a graphical summary of standardized path coefficients for each model. All three latent factors in Model 4 were highly correlated with one another; the correlation was 0.821 between the Burnout and STS factors, 0.770 between CF and STS factors, and 0.864 between CF and Burnout factors. Results of the same CFAs using the five hot-deck imputed datasets did not demonstrate any substantial differences.

**Table 2:** Model fit estimates from confirmatory factor analyses of five theoretical models of burnout, compassion fatigue, and secondary traumatic stress.

**Model**	**χ^2^**	***df***	**χ^2^/*df***	**TLI**	**SRMR**	**RMSEA(90% CI)**
1	2,583^*^	665	3.88	0.86	0.112	0.088 (0.084–0.091)
2	2,457^*^	664	3.70	0.87	0.108	0.085 (0.081–0.089)
3	2,307^*^	664	3.47	0.88	0.104	0.081 (0.078–0.085)
4	2,244^*^	662	3.39	0.89	0.103	0.080 (0.076–0.084)
5^a^	2,302^*^	664	3.47	0.88	0.102	0.081 (0.078–0.085)
**Model comparisons** ^**a**^	**Δχ^2^**	**Δ** ***df***		
2–1	−126^*^	1		
3–1	−276^*^	1		
4–2	−213^*^	2		
4–3	−63^*^	2		
4–1	−339^*^	3		

* *p < 0.001*.

a *Model 5 is not nested within previous models and therefore not compared by Δχ^2^. See Figure 1 for graphical depictions of Models 1–5*.

*CFI, Comparative Fit Index; TLI, Tucker-Lewis Index; SRMR, Standardized Root Mean Square Residual; RMSEA, Root Mean Square Error of Approximation; CI, confidence interval*.

### Exploratory Factor Analyses

In the absence of a factor model with acceptable performance on CFA, an EFA was conducted using the correlation matrix of data from CF, STS, and burnout scales. Kaiser-Meyer-Olkin measure of sampling adequacy was 0.930, and Barlett's test of sphericity was significant [$\chi_{\left(703\right)}^{2}$ = 7,340.9, *p* < 0.001], both of which suggested appropriateness of proceeding with the EFA. An initial unrotated PAF solution extracted seven factors with eigenvalues >1 [Eigenvalues (% of variance explained): 12.64 (33.3), 2.84 (7.5), 1.82 (4.8), 1.77 (4.7), 1.38 (3.6), 1.18 (3.1), and 1.14 (3.0), respectively]; however, only five of these factors are retained by Velicar's minimum average partial test, and only four factors by parallel analysis and scree test. Subsequent EFA was tested with five and four factors; ultimately, four factors were selected due to low primary factor loadings and high cross-loadings on a fifth extracted factor. The correlation matrix from the initial, unrotated PAF solution including all items are included in Supplementary Table 1.

Repeat EFA was set to extract four factors. Promax rotation was used to analyze interfactor correlations, which demonstrated substantial correlations between factors (range 0.212–0.633). Thus, an oblique rotation was deemed more appropriate than an orthogonal rotation, and promax was ultimately used in the final PAF solution. Investigation of factor pattern loadings revealed that a total of 11 items did not meet criteria for retention. Namely, item 21 of the ProQOL, item 1 of the BM-SV, and items 3, 5, 10, 13, and 15 of the STSS had secondary factor loadings >0.3; and items 7 and 8 of the BM-SV as well as items 2 and 12 of the STSS had primary factor loadings that were under 0.5. Items that were removed from the final solution and their primary and secondary factor loadings are outlined in Supplementary Table 2.

The pattern correlation matrix of the final four-factor solution using the remaining 27 items is represented in Table 3, along with the Eigenvalues and variance explained by each factor. The structure correlation matrix for the same EFA solution is included in Supplementary Table 3.

**Table 3:** Exploratory factor analysis of the BM-SV, ProQOL-21, and STSS: factor loadings from the pattern matrix of the final four-factor principal axis factoring promax-rotated solution.

**Scale item**	**Factor**				***h*2**
	**1**	**2**	**3**	**4**	
BM-SV4: Trapped	0.96				0.81
ProQOL10: I feel trapped by my job as a physician.	0.84				0.62
BM-SV3: Hopeless	0.77				0.68
BM-SV5: Helpless	0.77				0.67
BM-SV10: “I've had it”	0.75				0.61
BM-SV6: Depressed	0.65				0.59
ProQOL19 I feel worn out because of my work as a physician.	0.64				0.50
ProQOL11 Because of my medical practice, I have felt “on edge” about various things.	0.60				0.46
BM-SV2: Disappointed with people	0.57				0.50
ProQOL26 I feel “bogged down” by the system.	0.55				0.44
STSS7: I had little interest in being around others.		0.73			0.51
STSS8: I felt jumpy.		0.66			0.43
STSS14: I wanted to avoid working with some patients.		0.61			0.40
STSS6: Reminders of my work with patients upset me.		0.57	0.25		0.35
STSS16: I expected something bad to happen		0.56			0.37
STSS17: I noticed gaps in my memory about patient sessions.		0.56			0.45
STSS11: I had trouble concentrating.		0.55			0.36
STSS1: I felt emotionally numb.	0.25	0.55			0.35
STSS9: I was less active than usual.		0.54			0.47
ProQOL14: I feel as though I am experiencing the trauma of a patient.			0.64		0.37
ProQOL9: I think that I might have been affected by the traumatic stress of patients.			0.60		0.26
ProQOL13: I feel depressed because of the traumatic experiences of my patients.			0.57		0.25
ProQOL8: I am not as productive at work because I am losing sleep over traumatic experiences of a patient.			0.49		0.24
ProQOL25: As a result of my medical practice, I have intrusive, frightening thoughts.			0.47		0.20
ProQOL23: I avoid certain activities or situations because they remind me of frightening experiences of my patients.			0.46		0.33
BM-SV9: Difficulties sleeping				0.86	0.81
STSS4: I had trouble sleeping.				0.81	0.68
Eigenvalue	9.20	2.29	1.66	1.52	
% variance explained	34.1	8.5	6.1	5.6	
Cumulative % variance explained	34.1	42.5	48.7	54.3	

*Factor loadings <0.20 are suppressed*.

*BM-SV, Burnout Measure-Short Version; ProQOL, Professional Quality of Life Scale (though ProQOL-21 was used, item numbers are from original ProQOL); STSS, Secondary Traumatic Stress Scale; h2, communality*.

Items loading onto the first factor described feeling trapped, hopeless, helpless, worn out, depressed, “on edge,” “bogged down,” and disappointed, as measured by items from both the BM-SV and the ProQOL-21. Items from all three STSS subscales (seven from the Avoidance subscale, two from the Arousal subscale, and one from the Intrusion subscale) loaded onto the second factor, with “little interest in being around others” having the highest loading. Six ProQOL-21 items loaded onto the third factor; items pertained to emotional responses specifically relating to patients' traumatic experiences. One item from tbe BM-SV and one from the STSS-Arousal subscale loaded most highly onto the final factor. A summary of the four factors that emerged from EFA and their intercorrelations are described in Table 4.

**Table 4:** Descriptive statistics and intercorrelations for factors extracted from final exploratory principal axis factoring solution.

**Factor**	**No. items**	**Cronbach's alpha**	**Factor intercorrelations**			
			**1**	**2**	**3**	**4**
1. Depressive symptoms	8	0.892	1.00	0.64	0.41	0.35
2. PTS-like symptoms	9	0.851	0.64	1.00	0.53	0.41
3. Reactions to patients' trauma	6	0.707	0.41	0.53	1.00	0.33
4. Sleep disturbance	2	0.867	0.35	0.41	0.33	1.00

*Based on standardized item responses*.

*No., number; PTS, Primary Traumatic Stress*.

Repeat EFAs conducted using the five imputed datasets did not result in any appreciable qualitative differences.

## Discussion

While burnout, CF, and STS have been discussed at length, there is little consensus regarding their definitions. They have been variably described as three separate constructs, a single construct, or two constructs (with either burnout and CF or STS and CF representing a single underlying factor) (9, 12, 15–17, 22). None of these proposed theoretical factor structures are upheld by CFA in the present study of physicians. Mixed findings regarding relationships between CF, STS, and burnout in physicians may thus be attributable to a lack of construct validity that these terms and their associated measures suffer from in this population (10, 49, 50). In the absence of clearly delineated concepts, further attempts to discern correlations, influencing factors, and consequences of adverse physician experiences using existing tools will be limited by psychometric shortcomings and phenomenological gaps.

The present study's EFA is subject to the psychometric limitations of the measures used. As such, the factors that emerged from these analyses should not be interpreted as conclusive, alternative definitions of adverse physician experiences. Rather, examining the manner in which items from burnout, CF, and STS scales dissolve across dimensions that do not clearly correspond with any of the three concepts may offer insight into where and how existing constructs fracture. The items loading onto the first factor from EFA describes negative affective experiences related to work—feelings of stagnation, depletion, despondence. Rather than “burnout” or “compassion fatigue”, this factor seems more clearly summarized by the term “depressed mood”. Indeed, the distinction between burnout and depression, and whether this distinction is pragmatically or clinically relevant from a psychiatric standpoint, is a matter of its own contention, with recent literature suggesting that the two may not represent distinct phenomena (8). To define physicians' negative affective experiences, the relationships between these experiences and primary depression require clarification and consensus.

The second and third factors from EFA share common ground in their relationship to trauma. Items loading onto the second factor describe negative affective responses to patient care that mirror symptoms of primary traumatic stress, while those loading onto the third describe negative affective responses specific to patients' experiences of trauma. Thus, factors differ in terms of what appears to comprise the traumatic event: the patient care encounter or the patients' trauma. While prior definitions of STS have concatenated the two sets of responses into a single phenomenon, this analysis points toward the possibility of a difference (9–11). Separation of the data across these two different dimensions suggests that a physician's response to work that is symptomatically similar to primary traumatic stress may not necessarily represent a response to patients' experiences or narratives of primary trauma. Further investigations of PTS-like symptoms and physicians' responses to patients' trauma requires critical examination of various sources of distress physicians are exposed to and qualitative differences in their emotional, mental, and behavioral responses to them. The fourth factor emerging from EFA requires little interpretation, with both items loading onto it pertaining to sleep difficulties. To determine how insomnia fits into the scheme of adverse physician experiences, phenomenological clarity of these experiences, their roots, and their consequences must first be established.

If burnout, CF, STS are neither themselves nor one another, this begs the question: What are they? There is little doubt regarding the existence and prevalence of adverse physician experiences, though the terms used to describe these experiences may fall short of defining them. The failure of existing measures to capture clear factors underlying these terms may reflect the lack of theoretical consensus regarding these constructs, how they are related, and how they are different (9, 12, 14–22, 27, 28, 30–33). Alternatively, the lack of evidence for existing models in the present study may be attributable to the fact that the terms “burnout,” “CF,” and “STS” initially emerged to describe experiences of non-physician professionals (9, 29, 51). The lack of model fit for burnout, CF, and STS constructs observed in the present study thus cannot be generalized to non-physician healthcare providers, whose observable experiences may exhibit better fit with existing models and more discrimination between constructs. Given that existing quantitative measures emerged from constructs without consistent and demonstrable theoretical underpinnings, and that the terms “burnout,” “CF,” and “STS” initially emerged to describe non-physician professionals' experiences, further investigation of adverse physician experiences may benefit from characterization of potential underlying constructs using rigorous quantitative and/or open, generative qualitative approaches centering physicians. Through such investigations, cohesive descriptions and unifying constructs may be more clearly defined and provide a foundation for models of affective experiences among physicians. The ensuing clarity of terms and measures used to discuss and quantify negative affective physician experiences may then give way to a more robust pursuit of potential solutions.

Limitations of the present study include the relatively low response rate to the survey. This was anticipated in part, due to the low response rates of physicians in general, and because we could not implement incentives or additional participation-maximizing recruitment strategies due to resource constraints. However, the primary purpose of the study was not to survey and/or characterize the population, but rather to determine whether negative affective experiences were well-characterized by existing measures. A sample size of at least 300 was thus sought a priori to meet recommended power for factor analyses, and this goal was surpassed (39). Interpretation of the results should bear in mind that, by nature of the study design, results are subject to response bias. In our case, one way this may have manifested is a higher response rate from physicians who have a particular interest in burnout, CF, STS, and related topics.

An additional limitation of the study is that, to keep the survey at a reasonable length, we were only able to use one measure for each of the three constructs tested (burnout, CF, and STS). There are, however, multiple existing scales for each that we might have selected from. We selected the BM-SV, ProQOL-21, and STSS based on previous evidence that they performed well as unidimensional constructs (2, 3, 13). However, the unidimensionality of each construct was not upheld when the scales were examined in conjunction. It is unclear whether results would have varied should different scales had been used, and whether the problem of construct validity in the present study is a primarily psychometric one rather than a theoretical one with clear clinical implications. Further research should explore the replicability of these findings using different existing measures of burnout, CF, and STSS and in samples of different professional populations, such as nurses and psychotherapists. Additionally, the clinical implications of distinctions (or lack thereof) between burnout, CF, STS, and other pathologies in physicians are difficult to ascertain at this point, in the midst of conflicting theories and evidence. Future analyses of burnout, CF, STS, and related constructs in physicians should consider the clinical implications of symptom overlap and syndromic delineations.

## Conclusions

Existing models of burnout, compassion fatigue, and secondary traumatic stress constructs were not upheld by confirmatory factor analyses in our sample of Rhode Island allopathic physicians. Exploratory factor analysis revealed that responses varied along dimensions that do not clearly correspond with existing definitions of burnout, compassion fatigue, and secondary traumatic stress. Adverse affective experiences of physicians may not be well described by current conceptions of burnout, compassion fatigue, and secondary traumatic stress. Qualitative investigation based on physician narratives may guide identification of patterns and themes across adverse affective physician experiences.

## Data Availability Statement

The raw data supporting the conclusions of this article will be made available by the authors, without undue reservation.

## Ethics Statement

The studies involving human participants were reviewed and approved by Institutional Review Board of Brown University. Written informed consent for participation was not required for this study in accordance with the national legislation and the institutional requirements.

## Author Contributions

FA led the study, conducted analyses, and drafted the manuscript. JB and PA contributed to study design, data interpretation, and manuscript edits. All authors contributed to the article and approved the submitted version.

## Funding

This work was supported by the Bray Humanities Scholar Fellowship provided through the Warren Alpert Medical School of Brown University.

## Conflict of Interest

The authors declare that the research was conducted in the absence of any commercial or financial relationships that could be construed as a potential conflict of interest.

## Publisher's Note

All claims expressed in this article are solely those of the authors and do not necessarily represent those of their affiliated organizations, or those of the publisher, the editors and the reviewers. Any product that may be evaluated in this article, or claim that may be made by its manufacturer, is not guaranteed or endorsed by the publisher.

## References

[1] Schaufeli WB Taris TW. The conceptualization and measurement of burnout: common ground and worlds apart. Work Stress. (2005) 19:256–62. 10.1080/02678370500385913

[2] Malach-Pines A. The burnout measure, short version. Int J Stress Manag. (2005) 12:78–88. 10.1037/1072-5245.12.1.78

[3] Maslach C Leiter MP Schaufeli W. Measuring Burnout. Oxf Handb Organ Well Being (2008). Available online at: http://www.oxfordhandbooks.com/view/10.1093/oxfordhb/9780199211913.001.0001/oxfordhb-9780199211913-e-005 (accessed November 13, 2018).

[4] Malakh-Pines A Aronson E. Career Burnout: Causes and Cures. New York: Free Press (1988). p. 280.

[5] Maslach C Schaufeli WB Leiter MP. Job burnout. Annu Rev Psychol. (2001) 52:397–422. 10.1146/annurev.psych.52.1.397 11148311

[6] Maslach C. Burnout: The Cost of Caring. Cambridge, MA: ISHK (2003). p. 302.

[7] Maslach C. Job burnout: new directions in research and intervention. Curr Dir Psychol Sci. (2003) 12:189–92. 10.1111/1467-8721.01258

[8] Bianchi R. Do burnout and depressive symptoms form a single syndrome? Confirmatory factor analysis and exploratory structural equation modeling bifactor analysis. J Psychosom Res. (2020) 131:109954. 10.1016/j.jpsychores.2020.109954 32036062

[9] Figley CR. Compassion fatigue as secondary traumatic stress disorder: An overview. In: Figley CR, editor. Compassion Fatigue: Secondary Traumatic Stress Disorders from Treating the Traumatized. New York, NY: Brunner/ Mazel (1995). p. 1–20.

[10] Baird K Kracen A. Vicarious traumatization and secondary traumatic stress: a research synthesis. Couns Psychol Q. (2006) 19:181–8. 10.1080/09515070600811899

[11] Sprang G Craig C. An inter-battery exploratory factor analysis of primary and secondary traumatic stress: determining a best practice approach to assessment. Best Pract Ment Health. (2015) 11:1–13. Available online at: https://link.gale.com/apps/doc/A439635925/HRCA?u=anon~1c97e021&sid=googleScholar&xid=f5fbd430

[12] Stamm BH. The Concise ProQOL Manual. Pocatello (2010). Available online at: ProQOL.org (accessed February 28, 2022).

[13] Heritage B Rees CS Hegney DG. The ProQOL-21: a revised version of the professional quality of life (ProQOL) scale based on Rasch analysis. PLoS ONE. (2018) 13:e0193478. 10.1371/journal.pone.0193478 29489875 PMC5831102

[14] Lynch SH Lobo ML. Compassion fatigue in family caregivers: a Wilsonian concept analysis. J Adv Nurs. (2012) 68:2125–34. 10.1111/j.1365-2648.2012.05985.x 22435873

[15] Boscarino JA Figley CR Adams RE. Compassion fatigue following the September 11 terrorist attacks: a study of secondary trauma among New York City social workers. Int J Emerg Ment Health. (2004) 6:57–66. Available online at: https://www.ncbi.nlm.nih.gov/pmc/articles/PMC2713725/15298076 PMC2713725

[16] El-bar N Levy A Wald HS Biderman A. Compassion fatigue, burnout and compassion satisfaction among family physicians in the Negev area - a cross-sectional study. Isr J Health Policy Res. (2013) 2:31. 10.1186/2045-4015-2-31 23947591 PMC3765463

[17] Sorenson C Bolick B Wright K Hamilton R. Understanding compassion fatigue in healthcare providers: a review of current literature. J Nurs Scholarsh Off Publ Sigma Theta Tau Int Honor Soc Nurs. (2016) 48:456–65. 10.1111/jnu.12229 27351469

[18] Adams RE Boscarino JA Figley CR. Compassion fatigue and psychological distress among social workers: a validation study. Am J Orthopsychiatry. (2006) 76:103–8. 10.1037/0002-9432.76.1.103 16569133 PMC2699394

[19] Bride BE Radey M Figley CR. Measuring compassion fatigue. Clin Soc Work J. (2007) 35:155–63. 10.1007/s10615-007-0091-7

[20] Najjar N Davis LW Beck-Coon K Carney Doebbeling C. Compassion fatigue: a review of the research to date and relevance to cancer-care providers. J Health Psychol. (2009) 14:267–77. 10.1177/1359105308100211 19237494

[21] Meadors P Lamson A Swanson M White M Sira N. Secondary traumatization in pediatric healthcare providers: compassion fatigue, burnout, and secondary traumatic stress. Omega 2009. (2010) 60:103–28. 10.2190/OM.60.2.a 20222232

[22] Keidel GC. Burnout and compassion fatigue among hospice caregivers. Am J Hosp Palliat Med. (2002) 19:200–5. 10.1177/104990910201900312 12026044

[23] Aycock N Boyle D. Interventions to manage compassion fatigue in oncology nursing. Clin J Oncol Nurs. (2009) 13:183–91. 10.1188/09.CJON.183-191 19349265

[24] Sabo BM. Compassion fatigue and nursing work: can we accurately capture the consequences of caring work? Int J Nurs Pract. (2006) 12:136–42. 10.1111/j.1440-172X.2006.00562.x 16674780

[25] Weintraub AS Geithner EM Stroustrup A Waldman ED. Compassion fatigue, burnout and compassion satisfaction in neonatologists in the US. J Perinatol. (2016) 36:1021–6. 10.1038/jp.2016.121 27490191

[26] Wu S Singh-Carlson S Odell A Reynolds G Su Y. Compassion fatigue, burnout, and compassion satisfaction among oncology nurses in the United States and Canada. Oncol Nurs Forum. (2016) 43:E161–9. 10.1188/16.ONF.E161-E169 27314199

[27] Rourke MT. Compassion fatigue in pediatric palliative care providers. Pediatr Clin North Am. (2007) 54:631–44. 10.1016/j.pcl.2007.07.004 17933615

[28] Mcholm F. Rx for compassion fatigue. J Christ Nurs. (2006) 23:12–9. 10.1097/00005217-200611000-00003 17078229

[29] Figley CR. Compassion fatigue: psychotherapists' chronic lack of self care. J Clin Psychol. (2002) 58:1433–41. 10.1002/jclp.10090 12412153

[30] Maytum JC Heiman MB Garwick AW. Compassion fatigue and burnout in nurses who work with children with chronic conditions and their families. J Pediatr Health Care. (2004) 18:171–9. 10.1016/j.pedhc.2003.12.005 15224041

[31] Udipi S Veach PM Kao J LeRoy BS. The psychic costs of empathic engagement: personal and demographic predictors of genetic counselor compassion fatigue. J Genet Couns. (2008) 17:459–71. 10.1007/s10897-008-9162-3 18704664

[32] Abendroth M Flannery J. Predicting the risk of compassion fatigue: a study of hospice nurses. J Hosp Palliat Nurs. (2006) 8:346–56. 10.1097/00129191-200611000-00007

[33] Collins S Long A. Too tired to care? The psychological effects of working with trauma. J Psychiatr Ment Health Nurs. (2003) 10:17–27. 10.1046/j.1365-2850.2003.00526.x 12558918

[34] Klein CJ Riggenbach-Hays JJ Sollenberger LM Harney DM McGarvey JS. Quality of life and compassion satisfaction in clinicians: a pilot intervention study for reducing compassion fatigue. Am J Hosp Palliat Med. (2018) 35:882–8. 10.1177/1049909117740848 29169248

[35] Bourassa DB. Compassion fatigue and the adult protective services social worker. J Gerontol Soc Work. (2009) 52:215–29. 10.1080/01634370802609296 19308828

[36] Gribben JL MacLean SA Pour T Waldman ED Weintraub AS. A cross-sectional analysis of compassion fatigue, burnout, and compassion satisfaction in pediatric emergency medicine physicians in the United States. Acad Emerg Med. (2019) 26:732–43. 10.1111/acem.13670 31204794

[37] Salston M Figley CR. Secondary traumatic stress effects of working with survivors of criminal victimization. J Trauma Stress. (2003) 16:167–74. 10.1023/A:1022899207206 12699204

[38] Sodeke-Gregson EA Holttum S Billings J. Compassion satisfaction, burnout, and secondary traumatic stress in UK therapists who work with adult trauma clients. Eur J Psychotraumatology. (2013) 4:21869. 10.3402/ejpt.v4i0.21869 24386550 PMC3877781

[39] Wilson Van Voorhis CR Morgan BL. Understanding power and rules of thumb for determining sample sizes. Tutor Quant Methods Psychol. (2007) 3:43–50. 10.20982/tqmp.03.2.p043

[40] Bride BE Robinson MM Yegidis B Figley CR. Development and validation of the secondary traumatic stress scale. Res Soc Work Pract. (2004) 14:27–35. 10.1177/1049731503254106

[41] Sowden JF Schonfeld IS Bianchi R. Are Australian teachers burned-out or depressed? A confirmatory factor analytic study involving the Occupational Depression Inventory. J Psychosom Res. (2022) 157:110783. 10.1016/j.jpsychores.2022.110783 35325775

[42] Henson RK Roberts JK. Use of exploratory factor analysis in published research: common errors and some comment on improved practice. Educ Psychol Meas. (2006) 66:393–416. 10.1177/0013164405282485

[43] Bianchi R Schonfeld IS Verkuilen J. A five-sample confirmatory factor analytic study of burnout-depression overlap. J Clin Psychol. (2020) 76:801–21. 10.1002/jclp.22927 31926025

[44] Rex B. Kline. Principles and Practice of Structural Equation Modeling Fourth. New York, NY: The Guilford Press (2016).

[45] Werne C Schermelleh-Enge K. Deciding between Competing Models-Chi-Square Difference Tests. Available online at: http://www.psychologie.uzh.ch/dam/jcr:ffffffff-b371-2797-0000-00000fda8f29/chisquare_diff_en.pdf (accessed January 21, 2022).

[46] Cranmer SJ Gill J. We have to be discrete about this: a non-parametric imputation technique for missing categorical data. Br J Polit Sci. (2013) 43:425–49. 10.1017/S0007123412000312

[47] Rosseel Y. lavaan: an R package for structural equation modeling. J Stat Softw. (2012) 48:1–36. 10.18637/jss.v048.i0225601849

[48] O'Connor BP. SPSS and SAS programs for determining the number of components using parallel analysis and velicer's MAP test. Behav Res Methods Instrum Comput J Psychon Soc Inc. (2000) 32:396–402. 10.3758/BF03200807 11029811

[49] Smart D English A James J Wilson M Daratha KB Childers B. Compassion fatigue and satisfaction: a cross-sectional survey among US healthcare workers. Nurs Health Sci. (2014) 16:3–10. 10.1111/nhs.12068 23663318

[50] Manning-Jones S de Terte I. Secondary traumatic stress, vicarious posttraumatic growth, and coping among health professionals. A comparison study. J Psychol. (2016) 45:10. Available online at: https://www.psychology.org.nz/journal-archive/Secondary-Traumatic-Stress.pdf

[51] Leone SS Wessely S Huibers MJH Knottnerus JA Kant Ij. Two sides of the same coin? On the history and phenomenology of chronic fatigue and burnout. Psychol Health. (2011) 26:449–64. 10.1080/08870440903494191 20437294

