# Supplementary material for: Examining the Constructs of Burnout, Compassion Fatigue, Secondary Traumatic Stress in Physicians Using Factor Analyses
Source: Front Public Health. 2022 May 6;10:893165. doi: 10.3389/fpubh.2022.893165 (PMC9120964; doi:10.3389/fpubh.2022.893165)
Supplement: Supplementary file 1 [file Table_1.DOCX]

Supplemental Table 1: Correlation matrix from initial, unrotated exploratory factor analysis of burnout, compassion fatigue, and secondary traumatic stress items.

|  | PQ8 | PQ9 | PQ10 | PQ11 | PQ13 | PQ14 | PQ19 | PQ21 | PQ23 | PQ25 | PQ26 | BM1 | BM2 | BM3 | BM4 | BM5 | BM6 | BM7 | BM8 | BM9 | BM10 | STS1 | STS2 | STS3 | STS4 | STS5 | STS6 | STS7 | STS8 | STS9 | STS10 | STS11 | STS12 | STS13 | STS14 | STS15 | STS16 | STS17 |
| --- | --- | --- | --- | --- | --- | --- | --- | --- | --- | --- | --- | --- | --- | --- | --- | --- | --- | --- | --- | --- | --- | --- | --- | --- | --- | --- | --- | --- | --- | --- | --- | --- | --- | --- | --- | --- | --- | --- |
| PQ8 | 1.0 | .37 | .10 | .21 | .33 | .21 | .15 | .23 | .19 | .25 | .13 | .13 | .15 | .22 | .18 | .14 | .19 | .21 | .17 | .21 | .19 | .08 | .33 | .17 | .21 | .17 | .30 | .10 | .15 | .13 | .22 | .18 | .13 | .29 | .13 | .15 | .19 | .11 |
| PQ9 | .37 | 1.0 | .21 | .30 | .46 | .36 | .20 | .26 | .24 | .30 | .24 | .25 | .23 | .39 | .26 | .31 | .24 | .27 | .30 | .24 | .26 | .21 | .36 | .26 | .21 | .24 | .43 | .22 | .33 | .26 | .39 | .38 | .23 | .21 | .24 | .29 | .34 | .22 |
| PQ10 | .10 | .21 | 1.0 | .59 | .14 | .07 | .53 | .43 | .10 | .08 | .45 | .41 | .40 | .55 | .70 | .56 | .54 | .30 | .33 | .23 | .58 | .39 | .37 | .07 | .18 | .53 | .29 | .31 | .30 | .30 | .24 | .32 | .22 | .07 | .29 | .47 | .36 | .07 |
| PQ11 | .21 | .30 | .59 | 1.0 | .19 | .10 | .55 | .54 | .23 | .26 | .45 | .46 | .32 | .46 | .54 | .48 | .50 | .34 | .45 | .32 | .51 | .30 | .43 | .08 | .28 | .50 | .34 | .29 | .39 | .29 | .33 | .35 | .21 | .24 | .30 | .48 | .36 | .13 |
| PQ13 | .33 | .46 | .14 | .19 | 1.0 | .41 | .17 | .20 | .18 | .18 | .12 | .17 | .22 | .26 | .20 | .27 | .28 | .23 | .29 | .27 | .22 | .17 | .28 | .28 | .21 | .18 | .37 | .21 | .23 | .13 | .26 | .28 | .21 | .24 | .17 | .28 | .31 | .19 |
| PQ14 | .21 | .36 | .07 | .10 | .41 | 1.0 | .05 | .06 | .26 | .25 | .00 | .06 | .10 | .15 | .12 | .15 | .14 | .21 | .08 | .13 | .13 | .07 | .25 | .31 | .10 | .10 | .24 | .14 | .23 | .03 | .14 | .10 | .18 | .17 | .07 | .11 | .22 | .05 |
| PQ19 | .15 | .20 | .53 | .55 | .17 | .05 | 1.0 | .65 | .07 | .11 | .50 | .58 | .36 | .41 | .51 | .44 | .43 | .24 | .32 | .31 | .57 | .34 | .30 | .04 | .28 | .44 | .31 | .18 | .30 | .27 | .35 | .35 | .20 | .18 | .28 | .42 | .27 | .11 |
| PQ21 | .23 | .26 | .43 | .54 | .20 | .06 | .65 | 1.0 | .16 | .16 | .55 | .60 | .33 | .44 | .52 | .46 | .43 | .25 | .27 | .35 | .58 | .29 | .31 | .07 | .29 | .43 | .30 | .18 | .27 | .27 | .37 | .38 | .21 | .22 | .27 | .41 | .30 | .17 |
| PQ23 | .19 | .24 | .10 | .23 | .18 | .26 | .07 | .16 | 1.0 | .32 | .04 | .09 | .08 | .20 | .20 | .21 | .17 | .29 | .11 | .09 | .09 | .01 | .14 | .30 | .01 | .10 | .18 | .19 | .19 | .13 | .06 | .07 | .32 | .16 | .09 | .08 | .15 | .12 |
| PQ25 | .25 | .30 | .08 | .26 | .18 | .25 | .11 | .16 | .32 | 1.0 | .05 | .14 | .16 | .21 | .23 | .19 | .16 | .14 | .21 | .24 | .16 | .10 | .17 | .18 | .16 | .14 | .24 | .13 | .24 | .06 | .20 | .19 | .13 | .18 | .14 | .18 | .25 | .16 |
| PQ26 | .13 | .24 | .45 | .45 | .12 | .00 | .50 | .55 | .04 | .05 | 1.0 | .49 | .36 | .38 | .47 | .42 | .40 | .24 | .23 | .24 | .53 | .29 | .27 | .03 | .22 | .47 | .26 | .23 | .26 | .26 | .31 | .36 | .19 | .11 | .30 | .36 | .28 | .20 |
| BM1 | .13 | .25 | .41 | .46 | .17 | .06 | .58 | .60 | .09 | .14 | .49 | 1.0 | .47 | .40 | .49 | .42 | .50 | .28 | .34 | .43 | .56 | .32 | .27 | .04 | .35 | .47 | .29 | .21 | .31 | .34 | .30 | .40 | .18 | .17 | .26 | .48 | .28 | .21 |
| BM2 | .15 | .23 | .40 | .32 | .22 | .10 | .36 | .33 | .08 | .16 | .36 | .47 | 1.0 | .52 | .52 | .48 | .50 | .27 | .35 | .26 | .46 | .26 | .26 | .13 | .19 | .37 | .20 | .22 | .25 | .34 | .20 | .28 | .22 | .21 | .29 | .44 | .32 | .15 |
| BM3 | .22 | .39 | .55 | .46 | .26 | .15 | .41 | .44 | .20 | .21 | .38 | .40 | .52 | 1.0 | .78 | .78 | .65 | .45 | .49 | .28 | .57 | .41 | .43 | .13 | .19 | .49 | .37 | .32 | .39 | .40 | .29 | .37 | .24 | .19 | .30 | .50 | .47 | .19 |
| BM4 | .18 | .26 | .70 | .54 | .20 | .12 | .51 | .52 | .20 | .23 | .47 | .49 | .52 | .78 | 1.0 | .74 | .71 | .39 | .45 | .32 | .67 | .41 | .38 | .15 | .23 | .52 | .31 | .34 | .34 | .40 | .30 | .40 | .25 | .15 | .29 | .50 | .47 | .20 |
| BM5 | .14 | .31 | .56 | .48 | .27 | .15 | .44 | .46 | .21 | .19 | .42 | .42 | .48 | .78 | .74 | 1.0 | .69 | .41 | .46 | .27 | .54 | .47 | .43 | .17 | .19 | .50 | .32 | .37 | .41 | .43 | .22 | .36 | .31 | .15 | .34 | .46 | .42 | .20 |
| BM6 | .19 | .24 | .54 | .50 | .28 | .14 | .43 | .43 | .17 | .16 | .40 | .50 | .50 | .65 | .71 | .69 | 1.0 | .43 | .55 | .42 | .58 | .43 | .37 | .15 | .30 | .54 | .35 | .45 | .44 | .48 | .25 | .38 | .23 | .22 | .33 | .51 | .46 | .26 |
| BM7 | .21 | .27 | .30 | .34 | .23 | .21 | .24 | .25 | .29 | .14 | .24 | .28 | .27 | .45 | .39 | .41 | .43 | 1.0 | .35 | .24 | .30 | .30 | .43 | .24 | .13 | .34 | .34 | .41 | .46 | .46 | .25 | .30 | .24 | .26 | .20 | .30 | .28 | .15 |
| BM8 | .17 | .30 | .33 | .45 | .29 | .08 | .32 | .27 | .11 | .21 | .23 | .34 | .35 | .49 | .45 | .46 | .55 | .35 | 1.0 | .38 | .29 | .30 | .32 | .19 | .31 | .41 | .37 | .35 | .41 | .35 | .28 | .39 | .16 | .27 | .22 | .38 | .43 | .21 |
| BM9 | .21 | .24 | .23 | .32 | .27 | .13 | .31 | .35 | .09 | .24 | .24 | .43 | .26 | .28 | .32 | .27 | .42 | .24 | .38 | 1.0 | .44 | .18 | .23 | .13 | .77 | .40 | .31 | .23 | .31 | .28 | .25 | .37 | .15 | .26 | .12 | .32 | .31 | .27 |
| BM10 | .19 | .26 | .58 | .51 | .22 | .13 | .57 | .58 | .09 | .16 | .53 | .56 | .46 | .57 | .67 | .54 | .58 | .30 | .29 | .44 | 1.0 | .39 | .36 | .13 | .31 | .55 | .33 | .30 | .33 | .34 | .31 | .36 | .24 | .19 | .29 | .53 | .41 | .16 |
| STS1 | .08 | .21 | .39 | .30 | .17 | .07 | .34 | .29 | .01 | .10 | .29 | .32 | .26 | .41 | .41 | .47 | .43 | .30 | .30 | .18 | .39 | 1.0 | .38 | .18 | .19 | .46 | .36 | .44 | .41 | .38 | .30 | .36 | .18 | .09 | .36 | .48 | .39 | .22 |
| STS2 | .33 | .36 | .37 | .43 | .28 | .25 | .30 | .31 | .14 | .17 | .27 | .27 | .26 | .43 | .38 | .43 | .37 | .43 | .32 | .23 | .36 | .38 | 1.0 | .33 | .21 | .36 | .45 | .34 | .56 | .30 | .35 | .34 | .31 | .27 | .33 | .35 | .36 | .08 |
| STS3 | .17 | .26 | .07 | .08 | .28 | .31 | .04 | .07 | .30 | .18 | .03 | .04 | .13 | .13 | .15 | .17 | .15 | .24 | .19 | .13 | .13 | .18 | .33 | 1.0 | .10 | .16 | .28 | .27 | .37 | .24 | .14 | .20 | .29 | .20 | .16 | .15 | .29 | .18 |
| STS4 | .21 | .21 | .18 | .28 | .21 | .10 | .28 | .29 | .01 | .16 | .22 | .35 | .19 | .19 | .23 | .19 | .30 | .13 | .31 | .77 | .31 | .19 | .21 | .10 | 1.0 | .45 | .29 | .26 | .31 | .22 | .28 | .32 | .09 | .27 | .11 | .29 | .27 | .19 |
| STS5 | .17 | .24 | .53 | .50 | .18 | .10 | .44 | .43 | .10 | .14 | .47 | .47 | .37 | .49 | .52 | .50 | .54 | .34 | .41 | .40 | .55 | .46 | .36 | .16 | .45 | 1.0 | .45 | .46 | .43 | .44 | .33 | .41 | .25 | .20 | .37 | .48 | .46 | .21 |
| STS6 | .30 | .43 | .29 | .34 | .37 | .24 | .31 | .30 | .18 | .24 | .26 | .29 | .20 | .37 | .31 | .32 | .35 | .34 | .37 | .31 | .33 | .36 | .45 | .28 | .29 | .45 | 1.0 | .46 | .54 | .30 | .45 | .43 | .31 | .29 | .41 | .40 | .47 | .32 |
| STS7 | .10 | .22 | .31 | .29 | .21 | .14 | .18 | .18 | .19 | .13 | .23 | .21 | .22 | .32 | .34 | .37 | .45 | .41 | .35 | .23 | .30 | .44 | .34 | .27 | .26 | .46 | .46 | 1.0 | .51 | .47 | .27 | .37 | .32 | .24 | .35 | .40 | .44 | .26 |
| STS8 | .15 | .33 | .30 | .39 | .23 | .23 | .30 | .27 | .19 | .24 | .26 | .31 | .25 | .39 | .34 | .41 | .44 | .46 | .41 | .31 | .33 | .41 | .56 | .37 | .31 | .43 | .54 | .51 | 1.0 | .45 | .33 | .44 | .37 | .35 | .33 | .40 | .48 | .29 |
| STS9 | .13 | .26 | .30 | .29 | .13 | .03 | .27 | .27 | .13 | .06 | .26 | .34 | .34 | .40 | .40 | .43 | .48 | .46 | .35 | .28 | .34 | .38 | .30 | .24 | .22 | .44 | .30 | .47 | .45 | 1.0 | .35 | .43 | .24 | .27 | .33 | .44 | .35 | .21 |
| STS10 | .22 | .39 | .24 | .33 | .26 | .14 | .35 | .37 | .06 | .20 | .31 | .30 | .20 | .29 | .30 | .22 | .25 | .25 | .28 | .25 | .31 | .30 | .35 | .14 | .28 | .33 | .45 | .27 | .33 | .35 | 1.0 | .51 | .22 | .34 | .33 | .42 | .37 | .26 |
| STS11 | .18 | .38 | .32 | .35 | .28 | .10 | .35 | .38 | .07 | .19 | .36 | .40 | .28 | .37 | .40 | .36 | .38 | .30 | .39 | .37 | .36 | .36 | .34 | .20 | .32 | .41 | .43 | .37 | .44 | .43 | .51 | 1.0 | .35 | .29 | .40 | .51 | .44 | .40 |
| STS12 | .13 | .23 | .22 | .21 | .21 | .18 | .20 | .21 | .32 | .13 | .19 | .18 | .22 | .24 | .25 | .31 | .23 | .24 | .16 | .15 | .24 | .18 | .31 | .29 | .09 | .25 | .31 | .32 | .37 | .24 | .22 | .35 | 1.0 | .36 | .37 | .28 | .33 | .31 |
| STS13 | .29 | .21 | .07 | .24 | .24 | .17 | .18 | .22 | .16 | .18 | .11 | .17 | .21 | .19 | .15 | .15 | .22 | .26 | .27 | .26 | .19 | .09 | .27 | .20 | .27 | .20 | .29 | .24 | .35 | .27 | .34 | .29 | .36 | 1.0 | .24 | .23 | .34 | .25 |
| STS14 | .13 | .24 | .29 | .30 | .17 | .07 | .28 | .27 | .09 | .14 | .30 | .26 | .29 | .30 | .29 | .34 | .33 | .20 | .22 | .12 | .29 | .36 | .33 | .16 | .11 | .37 | .41 | .35 | .33 | .33 | .33 | .40 | .37 | .24 | 1.0 | .48 | .44 | .32 |
| STS15 | .15 | .29 | .47 | .48 | .28 | .11 | .42 | .41 | .08 | .18 | .36 | .48 | .44 | .50 | .50 | .46 | .51 | .30 | .38 | .32 | .53 | .48 | .35 | .15 | .29 | .48 | .40 | .40 | .40 | .44 | .42 | .51 | .28 | .23 | .48 | 1.0 | .54 | .28 |
| STS16 | .19 | .34 | .36 | .36 | .31 | .22 | .27 | .30 | .15 | .25 | .28 | .28 | .32 | .47 | .47 | .42 | .46 | .28 | .43 | .31 | .41 | .39 | .36 | .29 | .27 | .46 | .47 | .44 | .48 | .35 | .37 | .44 | .33 | .34 | .44 | .54 | 1.0 | .44 |
| STS17 | .11 | .22 | .07 | .13 | .19 | .05 | .11 | .17 | .12 | .16 | .20 | .21 | .15 | .19 | .20 | .20 | .26 | .15 | .21 | .27 | .16 | .22 | .08 | .18 | .19 | .21 | .32 | .26 | .29 | .21 | .26 | .40 | .31 | .25 | .32 | .28 | .44 | 1.0 |

Abbreviations: STS, secondary traumatic stress scale; bm, Burnout Measure-Short Version; PQ, Professional Quality of Life Scale (item numbers correspond to original ProQOL, even though the ProQOL-21 was used).

Supplemental Table 2: Items excluded from final exploratory factor analysis due to pattern matrix cross-loadings >.3 or primary pattern matrix factor loadings < .5.

| **Scale** | | | Item | | 1º factor *(loading)* | 2º factor *(loading)* |
| --- | --- | --- | --- | --- | --- | --- |
|  | Subscale | |  |  |  |  |
| **ProQOL** | | |  |  |  |  |
|  | CF | | 21. | I feel overwhelmed because my workload seems endless | 1 *(.630)* | 4 *(.348)* |
| **BM-SV** | | | 1. | Tired | 1 *(.570)* | 4 *(.370)* |
|  |  | | 7. | Weak/Sickly | 2 *(.283)* | 1 *(.251)* |
|  |  | | 8. | Insecure/Like a failure | 1 *(.288)* | 2 *(.243)* |
| **STSS** | | |  |  |  |  |
|  | Intrusion | | 2. | My heart started pounding when I thought about my work with patients. | 2 *(.288)* | 3 *(.275)* |
|  | Intrusion | | 3. | It seemed as if I was reliving the trauma(s) experienced by my patient(s). | 3 *(.395)* | 2 *(.335)* |
|  | Avoidance | | 5. | I felt discouraged about the future. | 1 *(.419)* | 2 *(.355)* |
|  | Intrusion | | 10. | I thought about my work with patients when I didn't intend to. | 2 *(.377)* | 4 *(.313)* |
|  | Avoidance | | 12. | I avoided people, places, or things that reminded me of my work with clients | 2 (.423) | 3 (.166) |
|  | Intrusion | | 13. | I had disturbing dreams about my work with patients. | 2 *(.309)* | 4 *(.236)* |
|  | Arousal | | 15. | I was easily annoyed. | 2 *(.467)* | 1 *(.333)* |
|  | | Abbreviations: *ProQOL,* Professional Quality of Life (item number from the original ProQOL); *BM-SV* = Burnout Measure, Short Version; *STSS,* Secondary Traumatic Stress Scale; 1º, primary; 2º, secondary. | | | | |

| Supplemental Table 3. Exploratory factor analysis of the BM-SV, ProQOL-21, and STSS: Factor loadings from the structure matrix of the final four-factor promax principal axis factoring solution. | | | | |
| --- | --- | --- | --- | --- |
| Item | Factor | | | |
|  | 1 | 2 | 3 | 4 |
| BM-SV4 | .89 | .52 | .37 | .25 |
| BM-SV5 | .80 | .57 | .40 | .18 |
| BM-SV3 | .80 | .54 | .46 | .20 |
| BM-SV6 | .78 | .61 | .37 | .34 |
| BM-SV10 | .77 | .48 | .31 | .42 |
| ProQOL10 | .76 | .43 | .22 | .20 |
| ProQOL11 | .66 | .46 | .36 | .33 |
| ProQOL19 | .64 | .40 | .22 | .35 |
| BM-SV2 | .59 | .39 | .28 | .22 |
| ProQOL26 | .58 | .41 | .17 | .28 |
| STSS8 | .45 | .70 | .43 | .32 |
| STSS16 | .51 | .69 | .46 | .30 |
| STSS6 | .41 | .67 | .54 | .34 |
| STSS7 | .41 | .67 | .31 | .23 |
| STSS11 | .47 | .65 | .36 | .39 |
| STSS1 | .50 | .59 | .20 | .18 |
| STSS9 | .48 | .59 | .23 | .24 |
| STSS14 | .40 | .58 | .26 | .14 |
| STSS17 | .22 | .49 | .27 | .25 |
| ProQOL9 | .35 | .46 | .67 | .25 |
| ProQOL13 | .27 | .36 | .61 | .26 |
| ProQOL14 | .13 | .21 | .56 | .11 |
| ProQOL8 | .21 | .23 | .49 | .25 |
| ProQOL25 | .21 | .25 | .48 | .21 |
| ProQOL23 | .18 | .20 | .43 | .04 |
| BM-SV9 | .40 | .40 | .36 | .89 |
| STSS4 | .30 | .37 | .27 | .82 |
| Abbreviations: *BM-SV*, Burnout Measure-Short Version; *ProQOL*, Professional Quality of Life Scale (though ProQOL-21 was used, item numbers are from original ProQOL); *STSS*, Secondary Traumatic Stress Scale | | | | |
